# Distribution and Transport of CO_2_ in Hyperbranched Poly(ethylenimine)-Loaded MCM-41: A Molecular Dynamics Simulation Approach

**DOI:** 10.1021/acsami.3c07040

**Published:** 2023-09-08

**Authors:** Junhe Chen, Hyun June Moon, Kyung Il Kim, Ji Il Choi, Pavithra Narayanan, Miles A. Sakwa-Novak, Christopher W. Jones, Seung Soon Jang

**Affiliations:** † Computational NanoBio Technology Laboratory, School of Materials Science and Engineering, Georgia Institute of Technology, 771 Ferst Drive NW, Atlanta, Georgia 30332-0245, United States; ‡ School of Chemical & Biomolecular Engineering, Georgia Institute of Technology, 311 Ferst Drive NW, Atlanta, Georgia 30332-0100, United States; § Global Thermostat LLC, 10275 E106th Avenue, Brighton, Colorado 80601, United States; ∥ Strategic Energy Institute, Georgia Institute of Technology, Atlanta, Georgia 30332, United States

**Keywords:** CO_2_ capture, hyperbranched poly(ethylenimine), MCM-41, force field, molecular dynamics simulation

## Abstract

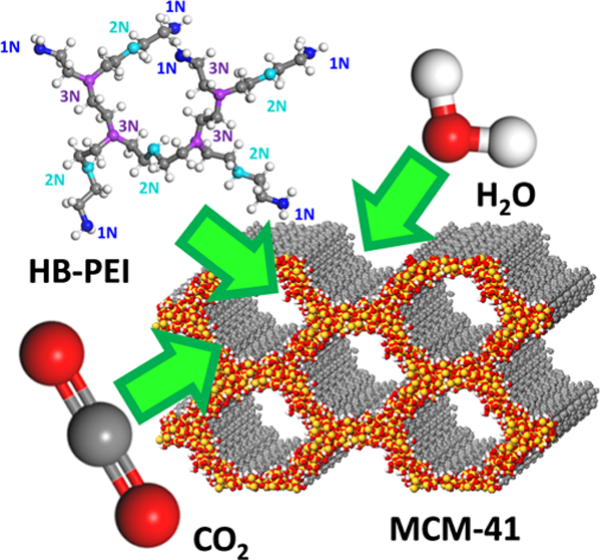

Fossil fuel use is accelerating climate change, driving the need for efficient CO_2_ capture technologies. Solid adsorption-based direct air capture (DAC) of CO_2_ has emerged as a promising mode for CO_2_ removal from the atmosphere due to its potential for scalability. Sorbents based on porous supports incorporating oligomeric amines in their pore spaces are widely studied. In this study, we investigate the intermolecular interactions and adsorption of CO_2_ and H_2_O molecules in hyperbranched poly(ethylenimine) (HB-PEI) functionalized MCM-41 systems to understand the distribution and transport of CO_2_ and H_2_O molecules. Density Functional Theory (DFT) is employed to compute the binding energies of CO_2_ and H_2_O molecules with HB-PEI and MCM-41 and to develop force field parameters for molecular dynamics (MD) simulations. The MD simulations are performed to examine the distribution and transport of CO_2_ and H_2_O molecules as a function of the HB-PEI content. The study finds that an HB-PEI content of approximately 34 wt % is thermodynamically favorable, with an upper limit of HB-PEI loading between 45 and 50 wt %. The distribution of CO_2_ and H_2_O molecules is primarily determined by their adsorptive binding energies, for which H_2_O molecules dominate the occupation of binding sites due to their strong affinity with silanol groups on MCM-41 and amine groups of HB-PEI. The HB-PEI content has a considerable impact on the diffusion of CO_2_ and H_2_O molecules. Furthermore, a larger number of water molecules (higher relative humidity) reduces the correlation of CO_2_ with the MCM-41 pore surface while enhancing the correlation of CO_2_ with the amine groups of the HB-PEI. Overall, the presence of H_2_O molecules increases the CO_2_ correlation with the amine groups and also the CO_2_ transport within HB-PEI-loaded MCM-41, meaning that the presence of H_2_O enhances the CO_2_ capture in the HB-PEI-loaded MCM-41. These findings are consistent with experimental observations of the impact of increasing humidity on CO_2_ capture while providing new, molecular-level explanations for the macroscopic experimental findings.

## 1 Introduction

The omnipresence of fossil fuels supplies an extensive amount of energy at the expense of climate change. The resulting emissions continue to raise the concentration of CO_2_ in the atmosphere at 2.3 ppm per year, calling attention to various technologies that can efficiently manage CO_2_ emissions and mitigate relevant environmental issues.^1^ Nonetheless, a significant separations challenge is presented by the atmospheric condition, in which the ratio of air to CO_2_ molecules is 2500 to 1.^2^

Researchers have recently investigated direct air capture (DAC) of CO_2_, specifically by utilizing selective sorption in liquids or on solids. Although the absorption approach is more commonly used in industry for CO_2_ separation, given the presence of aqueous media with a relatively high heat capacity, it is likely to require a large amount of heat to recover a meaningful amount of CO_2_.^3,4^ Conventional carbon capture and storage processes are energy-intensive. Thus, solid adsorption may be a more favorable option from an economic standpoint. Xu et al. further highlighted the advantage of the adsorption approach by noting its relatively low energy requirement and broad applicability across various temperature and pressure conditions.^5^ Similarly, numerous experimental studies with various adsorbents, such as activated carbons^6,7^ and metal oxides,^8,9^ have demonstrated the extensive applicability of adsorption for separating CO_2_ from gas mixtures. Various potential sorbents with high performance have also been the subject of simulation studies.^1,10−14^

To date, most studies describing DAC sorbent have focused on using solid-supported amine materials.^3,4,15−20^ Among these studies, solid amine sorbents based on porous oxide supports are the most common.^15^ A chemical reaction between CO_2_ and the amine functional group(s) occurs in supported amine materials, forming strong bonds leading to considerable uptakes, even at low CO_2_ partial pressures.^21^ As a result, the amine materials have superior uptakes associated with an elevated heat of sorption and selectivity toward CO_2_ compared to the physisorption-based sorbents such as zeolites and metal–organic frameworks.^15,22^ Although it has been demonstrated that the CO_2_ uptake in a wide range of mesostructured materials has a high correlation with the surface area of the materials,^23^ it should be noted that CO_2_ uptake of amine sorbents is primarily determined by the concentration of accessible amine functional groups rather than solely the surface area, especially when the CO_2_ concentration is less than 1 vol %.^15^

Various methods, such as physical impregnation, covalent tethering, in situ polymerization, and a combination of these approaches, have been employed to introduce amine moieties for functionalizing porous support materials. The impregnation approach, in which amine species are deposited into the pores or on the surface of the solid support, allows the amine moieties to be loaded without covalent bond forming chemical reactions with the surface.^15^ For instance, low-molecular-weight branched poly(ethylenimine) (PEI) has been employed in numerous studies as an amine-containing polymer to sorb CO_2_ because of its significant density of amine groups and good stability under temperature swing adsorption (TSA) or vacuum swing adsorption (VSA) conditions.^24,25^ In selecting different amines, Olah and co-workers stated that secondary amines were the best compromise between reactivity for CO_2_ uptake and energy for regeneration. Indeed, secondary amines have played major roles in industrial processes for acid gas separation since the 1980s.^26^ Typically, the CO_2_ uptake of the bare silica support is negligible under the same conditions due to the ultralow concentration of CO_2_.

Currently, the distribution and transport of CO_2_ molecules through specific capture materials have not been well elucidated by experimental methods due to the limited accessibility of useful techniques. For example, X-ray techniques face challenges because the inorganic solid supports overshadow organic amines and adsorbate species. NMR struggles with limited resolution in the solid state and complex interactions, making it difficult to provide clear insights. Lastly, neutron scattering is limited by its low neutron flux and the necessity of employing deuterated materials to achieve sufficient contrast. In this context, molecular dynamics (MD) simulation is a powerful tool that can provide molecular-level information on material systems of interest. Kim et al. discovered through MD simulation^1^ that the pair correlations of CO_2_ with the primary and secondary amines are reduced under hydrated conditions, suggesting that the carbamate formation mechanism is less prevalent compared to dry conditions. Regarding CO_2_ capture, Sharma and co-workers used MD simulation methods to determine which PEI nanostructures were conducive to CO_2_ capture, finding that free volume and entropy are the most influential factors in predicting the effectiveness of a given PEI.^27^ Another MD simulation study by Shen and co-workers demonstrated the importance of shortening the polymer chain length to increase CO_2_ capture.^28^ It is also reported that both primary and secondary amines in hyperbranched PEI (HB-PEI) strongly associate with CO_2_ molecules under dry conditions, while such CO_2_ association with the primary and secondary amines is reduced under hydrated conditions because the amine groups are associated with water molecules.^1^

Among diverse types of solid supports for constructing supported amine sorbents, ordered mesoporous silica materials, such as MCM-41, have demonstrated excellent CO_2_ capture performance with good thermal stability.^29^ They have tunable physical and chemical properties, such as pore surface functionalization and pore size,^30^ which provide ways to optimize the mass transfer of CO_2_ to sorption sites.^31^ Choi and co-workers impregnated commercial silica with 45% PEI and reached a CO_2_ uptake of 2.36 mmol/g from simulated ambient air (400 ppm of CO_2_ at room temperature).^32^ The Jones group and many others have explored a variety of approaches for incorporating amines into porous supports, such as grafting, impregnation, and in situ polymerization, for CO_2_ capture from simulated air.^15^ Researchers have loaded PEI into the nanosized pore channels of mesoporous silica supports and observed a significant increase in the CO_2_ adsorption capacity at the optimal amine loading (∼50 wt %), revealing that CO_2_ can form ammonium carbamates and ammonium bicarbonates with solid-supported amines but that tertiary amines are poor CO_2_ sorbents under the dry and humid conditions.^33−35^ Specifically, in solid sorbents, bicarbonate can be formed from primary, secondary, or tertiary amines but requires the presence of water, whereas alkylammonium carbamate or carbamic acid can be formed from primary and secondary amines under both dry and humid conditions.^36^

In this study, we investigate the distribution and transport of CO_2_ molecules through HB-PEI-loaded MCM-41 using the MD simulation method in the context of CO_2_ capture. For this purpose, we develop new force field parameters to describe the molecular interactions of CO_2_ and water with amine functional groups and silica surfaces. The distribution and transport of CO_2_ are characterized through pair correlation and mean square displacement analyses. Our study aids the understanding of CO_2_ capture in HB-PEI-loaded MCM-41 and provides insight into the design of new material systems with improved performance.

## 2 Models and Simulation Methods

### 2.1 Model Construction

**Figure 1:**
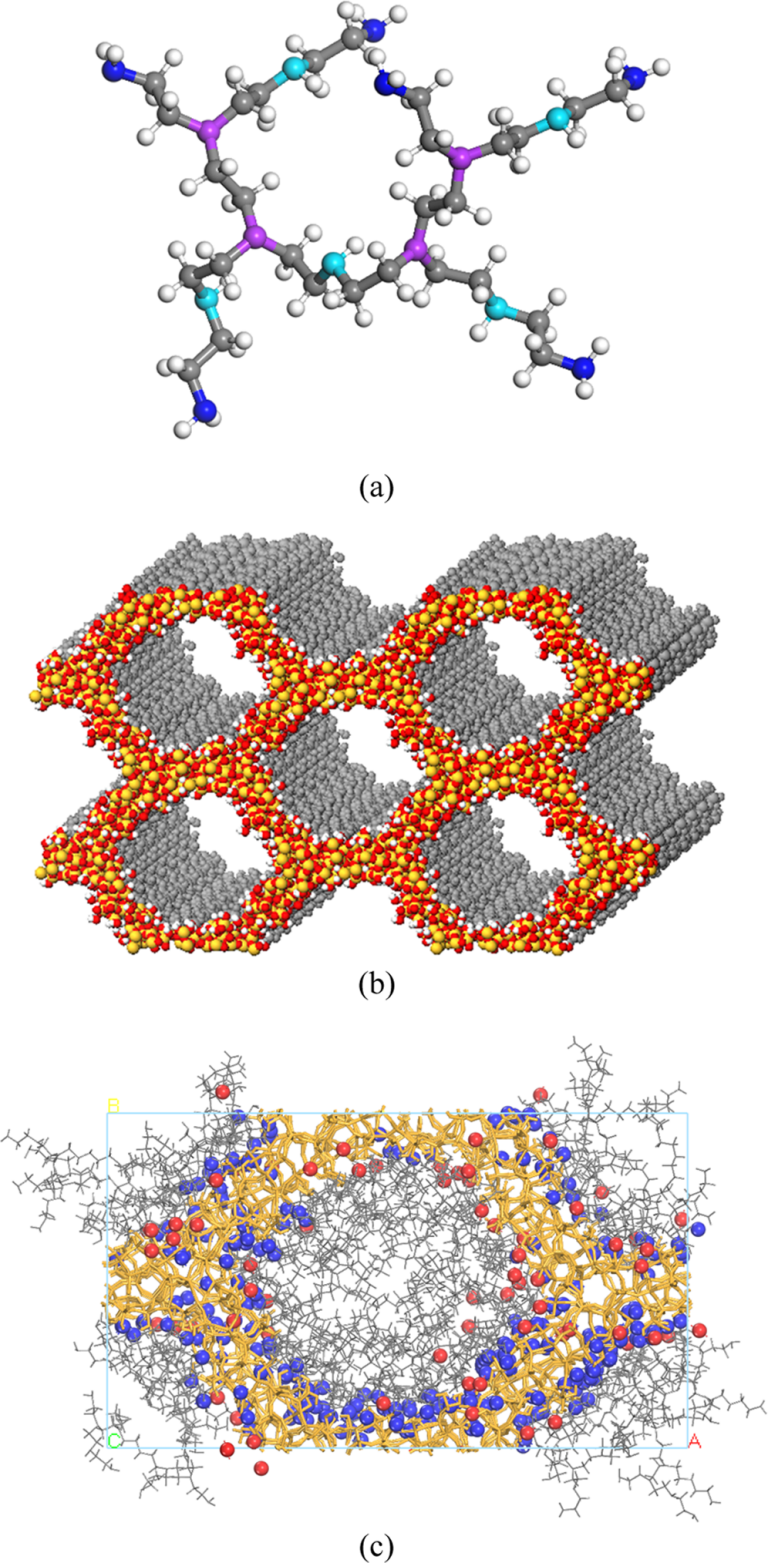
Atomic structures for (a) HB-PEI model; (b) MCM-41; and (c) integrated model of HB-PEI (20 wt %)-loaded MCM-41 with CO_2_ and H_2_O. In panel (a), the gray and white colors indicate carbon and hydrogen atoms, respectively, and the blue, cyan, and purple colors indicate primary, secondary, and tertiary amines, respectively. In panel (b), the orange and red colors indicate silicon and oxygen, respectively. In panel (c), the gray, blue, and red colors indicate HB-PEI, water, and CO_2_.

The molecular structure of HB-PEI was prepared as presented in Figure 1a, wherein the ratio of primary to secondary to tertiary amines was designed to be 6:5:4 (fitted to reproduce the NMR observation (42/33/25) and a molecular weight of 764.3).^1^ The molecular structure was geometrically optimized using the density functional theory (DFT) method with B3LYP and 6-31G** in Jaguar.^37^ Atomic charges were calculated by using Mulliken population analysis and assigned to individual atoms for molecular dynamics (MD) simulation. The structure of MCM-41 (Figure 1b) was employed from the previous study by Ugliengo and co-workers.^38^ Next, we introduced HB-PEI molecules into the pore of MCM-41 (Figure 1c), which is an example of the HB-PEI content of 20 wt %.

### 2.2 Force Fields for Molecular Interactions

In this study, we employed Emami’s force field,^39^ DREIDING force field,^40^ and F3C force field^41^ to describe the molecular interactions for MCM-41, HB-PEI, and water, respectively. DREIDING has been reported in numerous literatures to effectively describe the interactions of CO_2_ molecules (including in systems with amine or nitrogen), wherein the results have been validated against published or experimental data.^42−44^ The DREIDING force field has the following form:
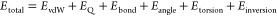
1where *E*_tot_, *E*_vdW_, *E*_Q_, *E*_bond_, *E*_angle_, *E*_torsion_, and *E*_inversion_ are the total, van der Waals, electrostatic, bond stretching, angle bending, torsion, and inversion energies, respectively. *E*_Q_ is calculated from atomic charges that are obtained from the Mulliken population analysis.

**Figure 2:**
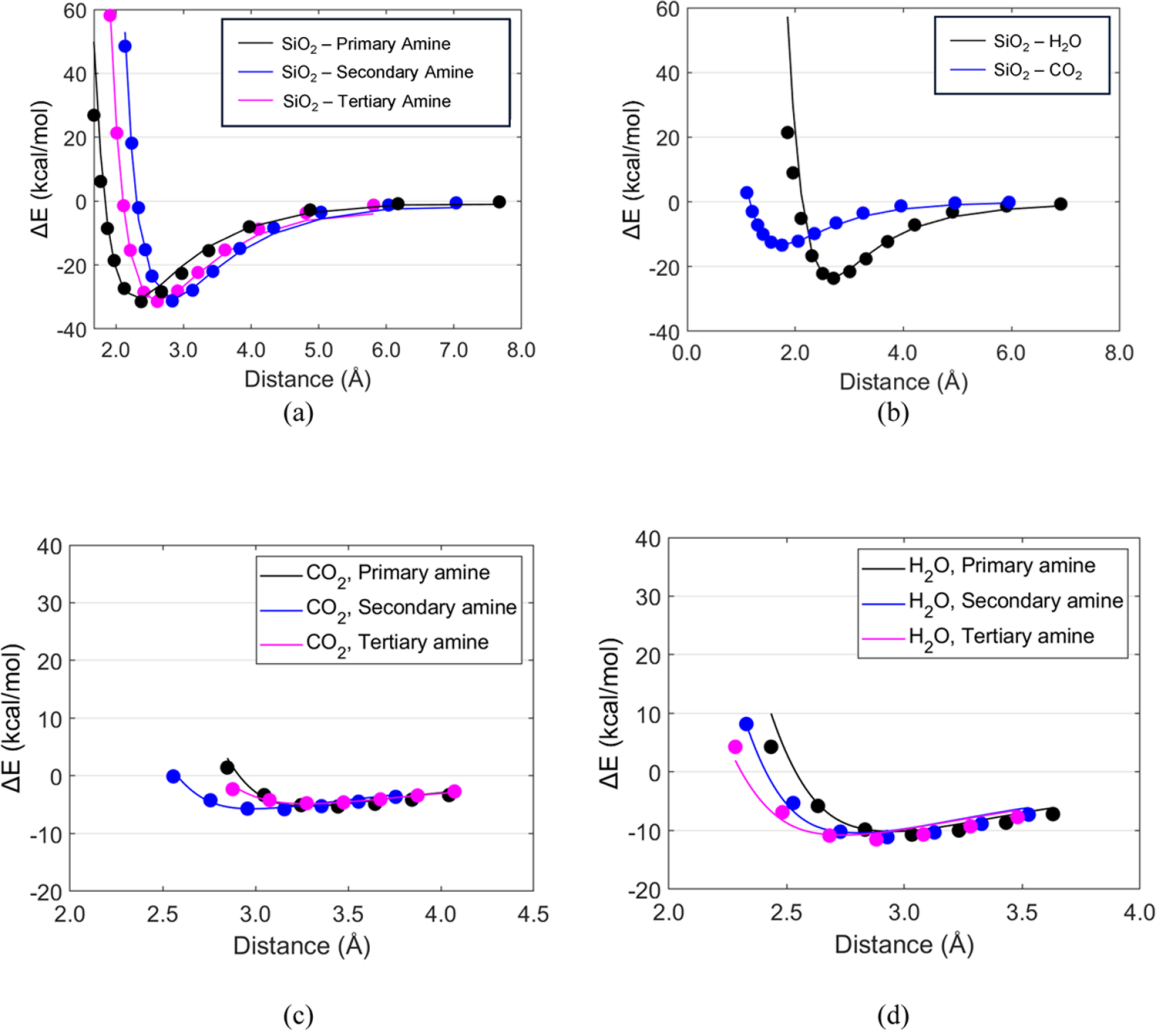
Binding energies calculated using newly developed force field parameters for off-diagonal van der Waals interaction (solid line) in comparison to those from DFT calculations (round circles): (a) SiO_2_–amines; (b) SiO_2_–water and CO_2_; (c) CO_2_–amines; and (d) H_2_O–amines.

To accurately investigate the heteromolecular interactions, we developed Lennard-Jones potential parameters of eq 2 for the off-diagonal van der Waals interactions to describe the interaction for amine-CO_2_, amine-H_2_O, amine-MCM-41, MCM-41-CO_2_, and MCM-41-H_2_O pairs
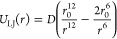
2We computed binding energy at various distances for amine- CO_2_, amine- H_2_O, amine-MCM-41, MCM-41-CO_2_, and MCM-41-H_2_O pairs using the DFT method and then determined the Lennard-Jones potential parameters using the least-squares method. Figure 2 shows a comparison of the binding energies calculated from the newly developed force field parameters with those from the DFT calculations, demonstrating that they are in good agreement. The overall mean square error has been reduced by approximately 98% when compared to the Lorentz–Berthelot standard mixing rule. Please note that the detailed parameters are summarized in Tables S1 and S2 in the Supporting Information, and the adsorption binding energies calculated using the standard mixing rule are included in Figure S1 in the Supporting Information.

### 2.3 Preparation of Three-Dimensional (3D) Systems

**Figure 3:**
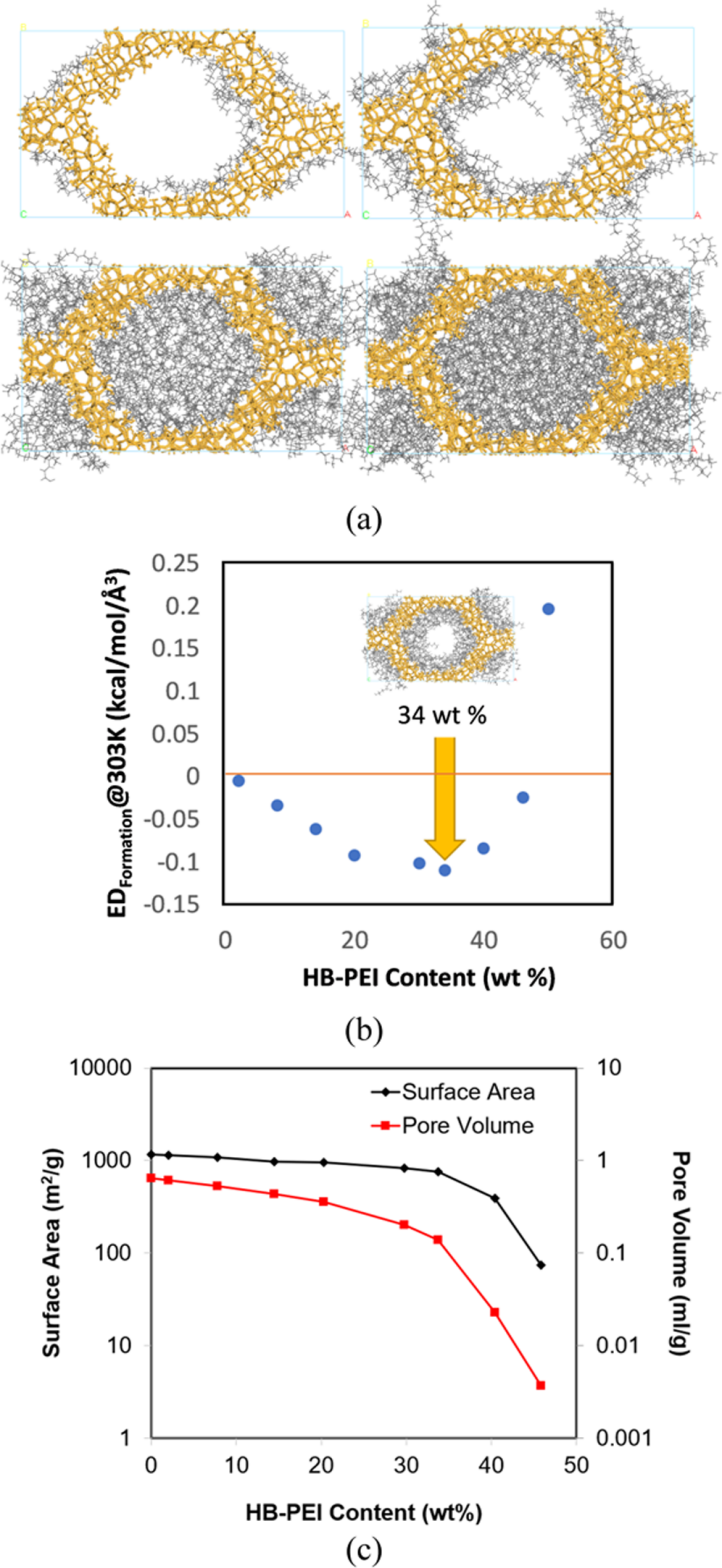
(a) Front views of optimized structures of HB-PEI-loaded MCM-41 with various HB-PEI contents such as 8, 20, 40, and 50%; (b) formation energy density as a function of HB-PEI content; and (c) surface area and pore volume of HB-PEI-loaded MCM-41 with different PEI loadings.

As shown in Figure 3a, we constructed 3D simulation systems of HB-PEI-loaded MCM-41 (PEI/MCM) with various HB-PEI contents, from which we identified the most stable HB-PEI content using the formation energy density at 303 K (ED_formation@303K_) defined by
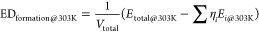
3where *E*_total@303K_ and *E*_*i*@303K_ denote the energy of the total system and the energy of the component *i*, respectively, at 303 K, and *V*_total_ and η_*i*_ denote the volume of the total system and the number of the component *i*, respectively. Figure 3b shows that ED_formation@303K_ has the minimum point at a HB-PEI content of 34 wt %.

Therefore, we constructed various simulation systems using an HB-PEI content of 34 wt % in MCM-41, as summarized in Table 1. In detail, PEI/MCM-34-0-0 has only the HB-PEI membrane in MCM-41 without CO_2_ and H_2_O molecules, whose main purpose is to characterize the distribution of primary, secondary, and tertiary amines of HB-PEI in the MCM-41. In this study, we simulated 72, 144, and 288 water molecules in PEI/MCM systems without CO_2_ to investigate the effect of hydration on the PEI/MCM system, while we simulated 12, 24, 36, 48, and 72 CO_2_ molecules in PEI/MCM systems without water to investigate the distribution and transport of CO_2_ molecules in the absence of moisture. Lastly, we simulated 72 CO_2_ molecules with 72 and 288 water molecules in the PEI/MCM system to investigate the effect of hydration on the amine-CO_2_ interaction.

**Table 1:** Configurations of the Simulated Systems^a^

systems	number of H_2_O	number of CO_2_
PEI/MCM-34-0-0	0	0
PEI/MCM-34-72-0	72	0
PEI/MCM-34-144-0	144	0
PEI/MCM-34-288-0	288	0
PEI/MCM-34-0-12	0	12
PEI/MCM-34-0-24	0	24
PEI/MCM-34-0-36	0	36
PEI/MCM-34-0-48	0	48
PEI/MCM-34-0-72	0	72
PEI/MCM-34-72-72	72	72
PEI/MCM-34-288-72	288	72

a HB-PEI content in MCM-41 = 34 wt % for system 1 to system 11.

In the simulated systems, we also analyzed the concentrations of CO_2_ and H_2_O by considering the pore volume. 72, 144, and 288 water molecules correspond to the concentrations of 0.078, 0.156, and 0.310 g/cm^3^, respectively, while 12, 24, 36, 48, and 72 CO_2_ molecules correspond to the concentrations of 0.031, 0.063, 0.094, 0.126, and 0.189 g/cm^3^, respectively. Here, it should be noted that 288 and 75 are not necessarily the maximum number of molecules for H_2_O and CO_2_, respectively.

### 2.4 Equilibration MD Simulations of HB-PEI-Loaded MCM-41

All MD simulations were performed in LAMMPS software.^45^ To reach equilibrium states within a reasonable amount of time, we utilized a thermal annealing procedure outlined by Jang and Goddard, which is used to accelerate the equilibration process by proving additional kinetic energy through repetitive thermal cycles.^45^ It is important to note that no particular structure for HB-PEI-loaded MCM-41 is assumed during the annealing procedure. Subsequently, a 200 ps NVT MD simulation and a 1 ns NPT MD simulation were conducted using a Nosé–Hoover thermostat to complete the annealing procedure. Finally, NPT MD simulations were performed for 30 ns at 303.15 K and 1 atm, and the systems in equilibrium with the last 10 ns in the trajectory files were used for analysis.

## 3 Results and Discussion

### 3.1 Force Field Development

Binding energies were calculated from the difference between the total energy of the molecular pair and the sum of the energies of each component molecule. Distance values were obtained by measuring the linear distance between the centroids of each molecule. The minimum energy value observed on the curve characterized the most stable distance between molecules. The agreement of the fitted curve with DFT values confirms that the newly developed FF parameters can reproduce the interactions described by DFT modeling. The binding energies of CO_2_ and amine (−5.31, −5.78, and −4.75 kcal/mol, for primary, secondary, and tertiary amine, respectively, in Figure 2c) are smaller than those of H_2_O and amine (−10.67, −11.1, and −11.5 kcal/mol, for primary, secondary, and tertiary amine, respectively, in Figure 2d). Among the CO_2_–amine pairs, the tertiary amine has the weakest bonding with CO_2_. However, the CO_2_-tertiary amine interaction becomes weaker due to the steric hindrances in HB-PEI. It is found that the SiO_2_-amine binding energy (Figure 2a) is the strongest compared to other binding energies (Figure 2b–2d), implying that CO_2_ and H_2_O might not be able to change the adsorption of HB-PEI on the MCM-41 surface. Please note that the interactions of H_2_O with SiO_2_ and amines are stronger than those of CO_2_ with SiO_2_ and amines, inferring that when the moisture content grows in the inflow air, CO_2_ will be captured less because H_2_O molecules may dominantly occupy the binding sites for CO_2_ capture.

### 3.2 HB-PEI Content in MCM-41

Figure 3a demonstrates the MD simulation results and the most probable structures of HB-PEI-loaded MCM-41 at different PEI weight loadings. From prior experimental work, when added to a bare support, PEI initially forms pore-coating layers, as shown in the small-angle neutron scattering study of Holewinski et al.^46^ The wall-coating PEI layers provide the most stable incorporation of PEI in the mesopores, and subsequent PEI molecules are then stacked on the layers of the PEI noncovalently bound to the walls. The pore surfaces of MCM-41 are nearly fully covered at ∼30 wt % HB-PEI content. In Figure 3b, the formation energy density reveals that 34 wt % HB-PEI content yields the minimal energy density, indicating that the PEI layers lined along the pore walls resulted in the most stable structure. Further introduction of PEI into the pores destabilizes the system, as indicated by the increased energy density of the systems. Consequently, we set up the potential limit of the HB-PEI loadings between 45 and 50 wt %, in which the formation energy density turns positive and energy minimization becomes almost unachievable at ∼50 wt % HB-PEI content. On the other hand, we found that the experimental studies in the literature pointed out that the upper limit of HB-PEI content could reach 60–75 wt %, with optimal CO_2_ uptake at ∼50 wt %.^34^ In our simulation, the average pore diameter is measured as 3.3 nm, with a surface area of 1160 m^2^/g and pore volume of 0.64 mL/g. Figure 3c shows the surface areas and pore volumes of HB-PEI-occupied MCM-41. Both the pore size and surface area decreased after HB-PEI was loaded to the MCM-41 channels. The pores were completely occupied by HB-PEI when the loading increased to 45–50%, agreeing well with the experimental data.^30^ The data align coherently with the structural configurations depicted in Figure 3a. A noted observation is the progressive decrement in pore volume corresponding to the escalating weight fraction of HB-PEI, peaking at a concentration of 34%. Beyond this threshold, the rate of decrease in pore volume intensifies with further increases in PEI loadings. It is plausible to hypothesize that initially, PEI establishes wall-coating domains up to a certain limit, and subsequent introduction of PEI tends to form aggregates, which exhibit lower stability compared to PEI deployed around the walls. At a PEI loading of 34 wt %, the decline in pore volume is 78.1%, with surface area witnessing a 35.1% reduction. These observations give insight into the dimensions of the wall-coating domain, which may differ here using MCM supports compared to the work of Hoelwinski et al., using SBA supports. We hypothesize that the realistic experimental systems may include structural defects,^47^ which may better accommodate higher PEI contents. In our current model, we established an infinite periodic MCM-41 supercell without any surface defects or deformation, which may not accurately represent realistic experimental systems. As a result, here, all PEI molecules are placed within the infinitely long channels of the MCM-41 structure. This assumption simplifies the system and allows us to focus on the interactions between PEI and the pore walls, neglecting the end effects at the pore mouth. However, it should be noted that real-world systems may have more complexity due to structural defects and polydispersity, such as different channel lengths or particle sizes. To improve the model, we suggest incorporating structural defects around the pore walls as they can potentially accommodate higher PEI contents, as seen in the experimental studies in the literature.

### 3.3 Distribution of CO_2_ and H_2_O in HB-PEI-Loaded MCM-41

**Figure 4:**
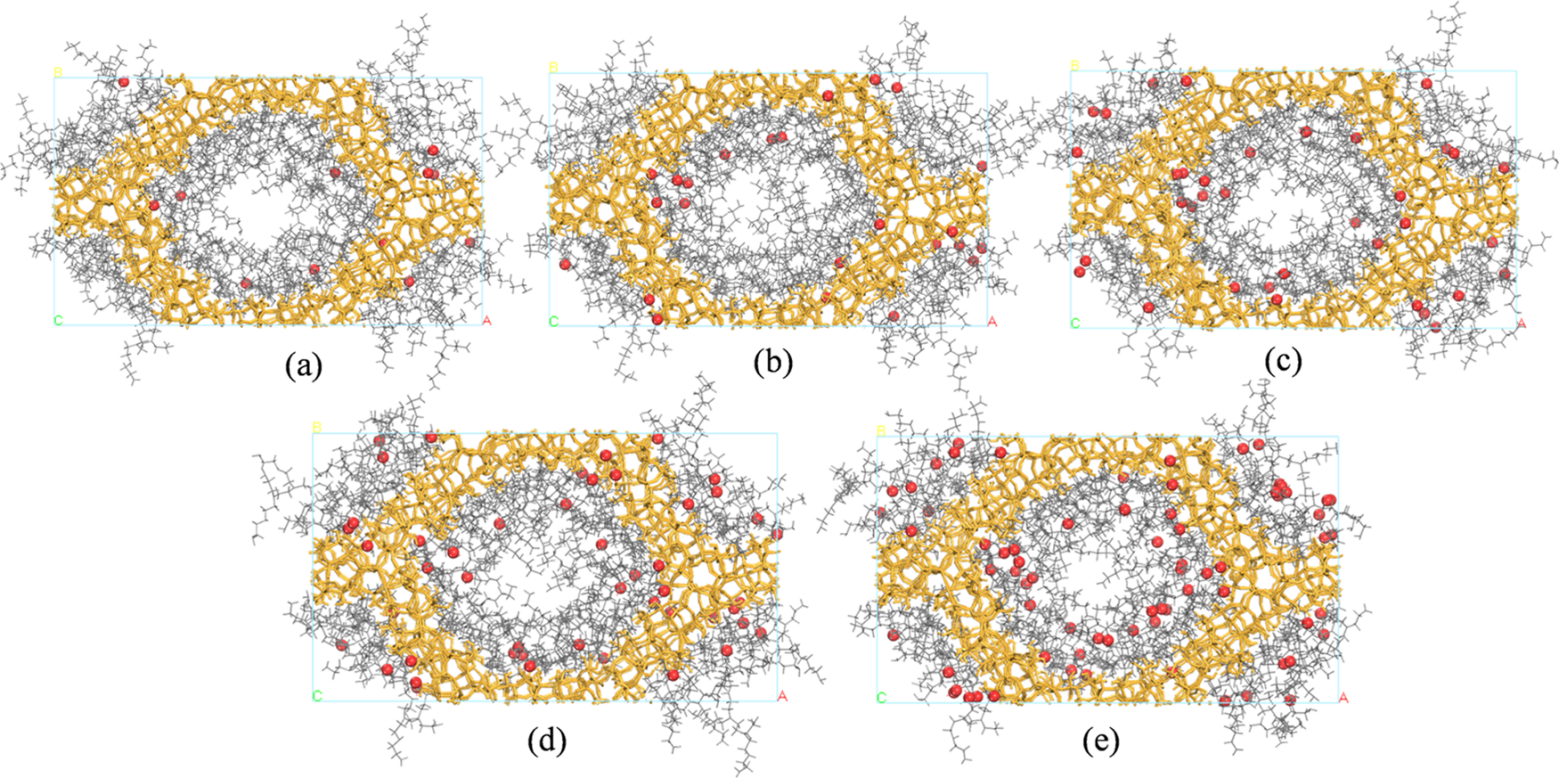
Distribution of CO_2_ molecules in 34 wt % HB-PEI-loaded MCM-41. The number of CO_2_ molecules is (a) 12 (PEI/MCM-34-0-12); (b) 24 (PEI/MCM-34-0-24); (c) 36 (PEI/MCM-34-0-36); (d) 48 (PEI/MCM-34-0-48); and (e) 72 (PEI/MCM-34-0-72). The orange, gray, and red colors indicate MCM-41, HB-PEI, and CO_2_.

**Figure 5:**
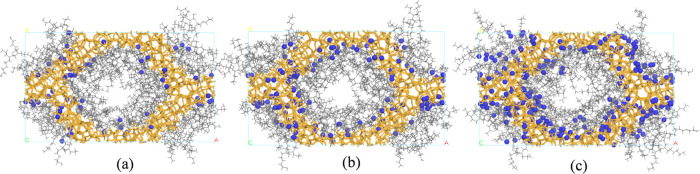
Distribution of H_2_O molecules in 34 wt % HB-PEI-loaded MCM-41. The number of H_2_O molecules is (a) 72 (PEI/MCM-34-72-0); (b) 144 (PEI/MCM-34-144-0); and (c) 288 (PEI/MCM-34-288-0). The orange, gray, and blue colors indicate MCM-41, HB-PEI, and water.

To investigate the distribution of CO_2_ and H_2_O, various configurations were simulated (as detailed in Table 1). Figures 4 and 5 reveal that under thermodynamically favorable HB-PEI content (34 wt %), the MCM-41 surface is fully covered by HB-PEI, while voids remain at the pore center for efficient molecular transport.

For systems with dry CO_2_ molecules (PEI/MCM-34-0-*N*, where the number of CO_2_ per pore *N* = 12, 24, 36, 48, and 72), low concentrations of CO_2_ molecules were observed in the vicinity of the MCM-41 surface (PEI/MCM-34-0-*N*, where *N* = 12, 24, and 36; see Figure 4a–4c). However, at higher concentrations (PEI/MCM-34-0-*N*, where *N* = 50 and 75; see Figure 4d,4e), CO_2_ molecules were also observed in the HB-PEI phase. In contrast, for systems with only H_2_O molecules (PEI/MCM-34-*M*-0, where *M* = 72, 144, and 288; see Figure 5), most of the H_2_O molecules were adsorbed on the MCM-41 surface, even under high hydration conditions (144 and 288 H_2_O molecules in the PEI/MCM system). This difference in the adsorption behavior of CO_2_ and H_2_O can be attributed to their respective adsorptive binding energies (−13.36 and −23.60 kcal/mol for SiO_2_–CO_2_ and SiO_2_–H_2_O, respectively, as shown in Figure 2b).

**Figure 6:**
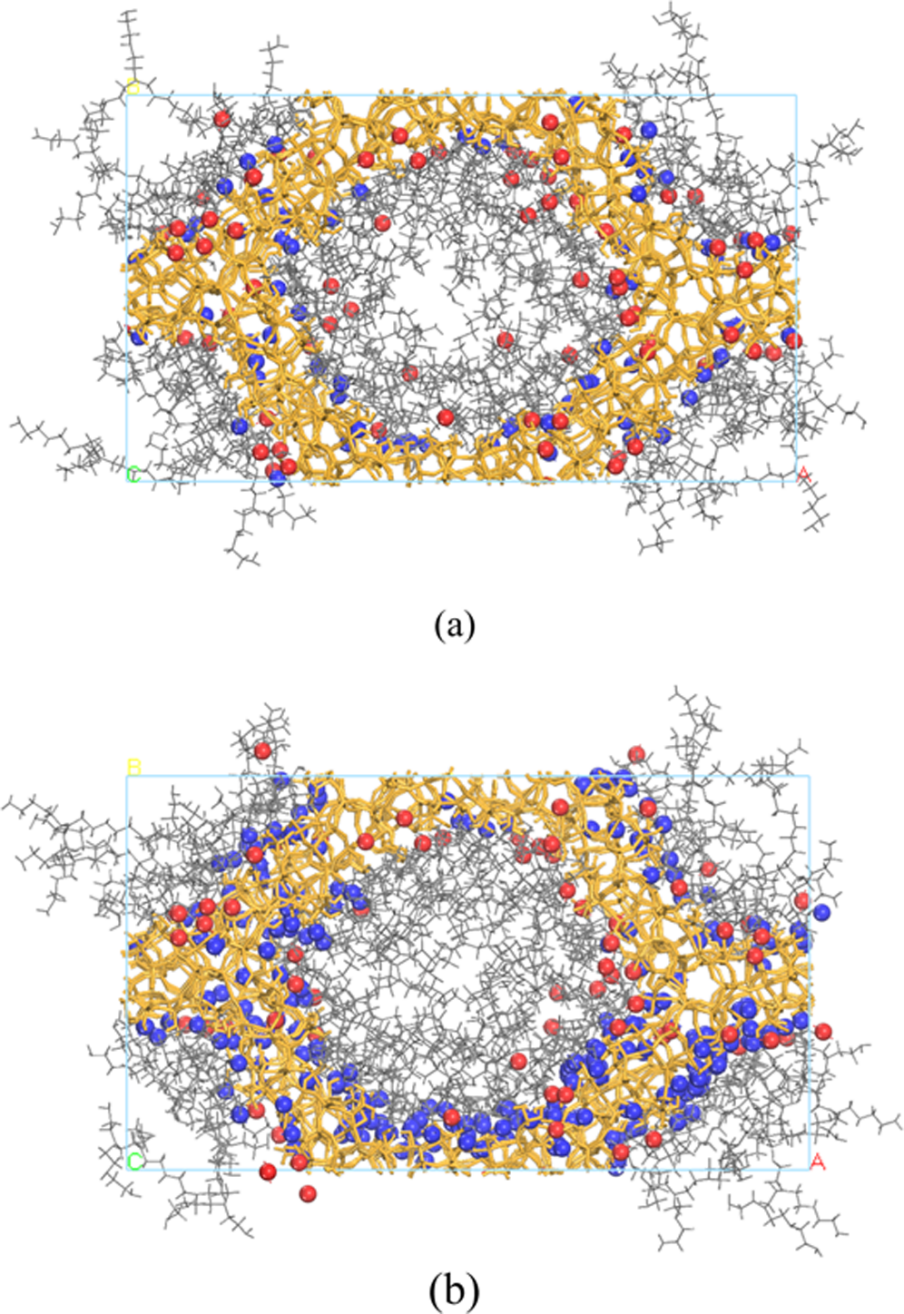
Distribution of CO_2_ and H_2_O in PEI/MCM with 34 wt % HB-PEI content: (a) 72 CO_2_ molecules and 72 H_2_O molecules (PEI/MCM-34-72-72); (b) 72 CO_2_ molecules and 288 H_2_O molecules (PEI/MCM-34-288-72). The orange, gray, red, and blue colors indicate MCM-41, HB-PEI, CO_2_, and water.

It can be hypothesized that once the binding sites, such as silanol groups on the pore surface of MCM-41, are saturated by CO_2_ and H_2_O molecules, the amine groups will associate with these molecules. In particular, it should be noted that H_2_O dominates due to its stronger binding energy with the silanol groups of MCM-41 and the amine groups of HB-PEI. To confirm the dominant effect of H_2_O on the distribution of CO_2_ and H_2_O molecules, we simulated a mixed H_2_O–CO_2_ gas in the PEI/MCM system with 34 wt % HB-PEI content (PEI/MCM-34-72-72 and PEI/MCM-34-288-72), as shown in Figure 6. Consistent with the results in Figures 4 and 5, CO_2_ and H_2_O molecules were primarily found near the MCM-41 pore surface due to their respective binding energies. These results suggest that the bicarbonate formation mechanism would likely occur near the MCM-41 pore surface due to the copresence of CO_2_ and H_2_O molecules, while the carbamate formation mechanism would be more available at the HB-PEI phase.

To quantitatively characterize the distribution of CO_2_ and H_2_O, we used the pair correlation function for the amine-CO_2_, MCM-41-CO_2_, amine-H_2_O, and MCM-41-H_2_O pairs. The pair correlation function, *g*_A–B_(*r*), represents the probability density of finding A and B atoms at a distance of r, averaged over the equilibrium trajectory, as shown in eq 4
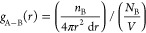
4where *n*_B_ refers to the number of B particles located at a distance *r* from particle A within a shell of thickness d*r*, *N*_B_ is the total number of B particles in the entire system, and *V* represents the volume of the entire system. Please note, however, that this definition in eq 4 is the ratio between the number densities of B particles in the shell and in the entire system. Thus, this *g*_A–B_(*r*) is a normalized unitless quantity that is converged to the value of 1.0 in most amorphous phases because the numerator and the denominator will become identical in a large value of *r*. Further, because *g*(*r*) is a normalized quantity, we cannot directly compare the magnitude of two different *g*(*r*). Therefore, to compare the pair correlation information quantitatively, we need to calculate un-normalized information.

For this, we move the number density of B particle  in eq 4 to the left-hand side and obtain eq 5
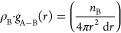
5Now, ρ_B_·*g*_A–B_(*r*) will have only the number density of the B particle at the distance *r* from the central A particle, and thereby, high number density and low number density can be directly interpreted to have strong and weak correlations, respectively.

**Figure 7:**
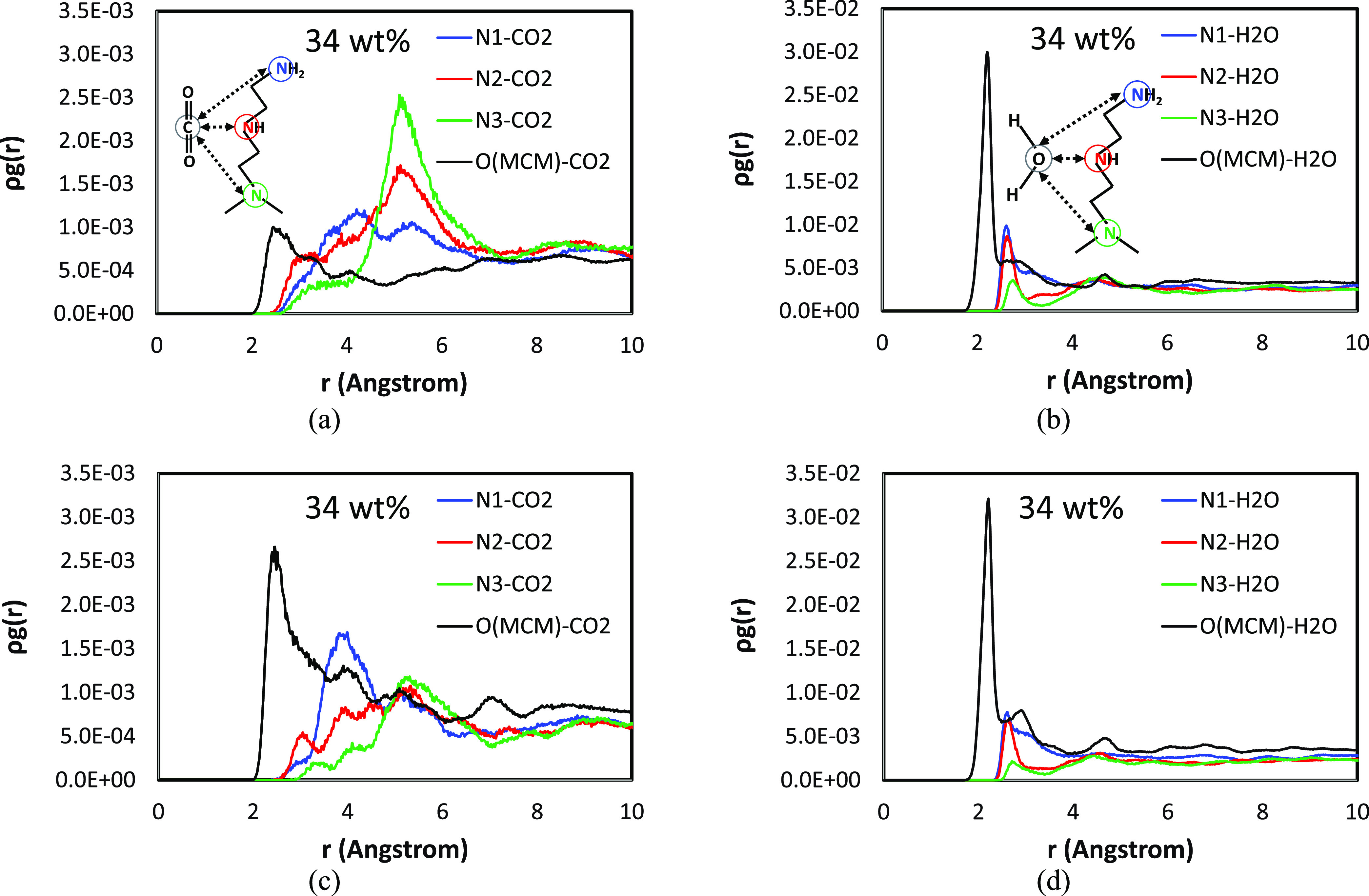
Pair correlation analysis in PEI/MCM systems with 34 wt % HB-PEI content: (a) amine-CO_2_ pair and MCM-41-CO_2_ for pure CO_2_ in PEI/MCM-34-0-72; (b) amine-H_2_O pair and MCM-41-H_2_O pair for pure H_2_O in PEI/MCM-34-288-0; (c) amine-CO_2_ pair and MCM-41-CO_2_ pair for CO_2_/H_2_O Mixed gas in PEI/MCM-34-288-72; and (d) amine-H_2_O pair and MCM-41-H_2_O pair for CO_2_/H_2_O Mixed gas in PEI/MCM-34-288-72.

**Figure 8:**
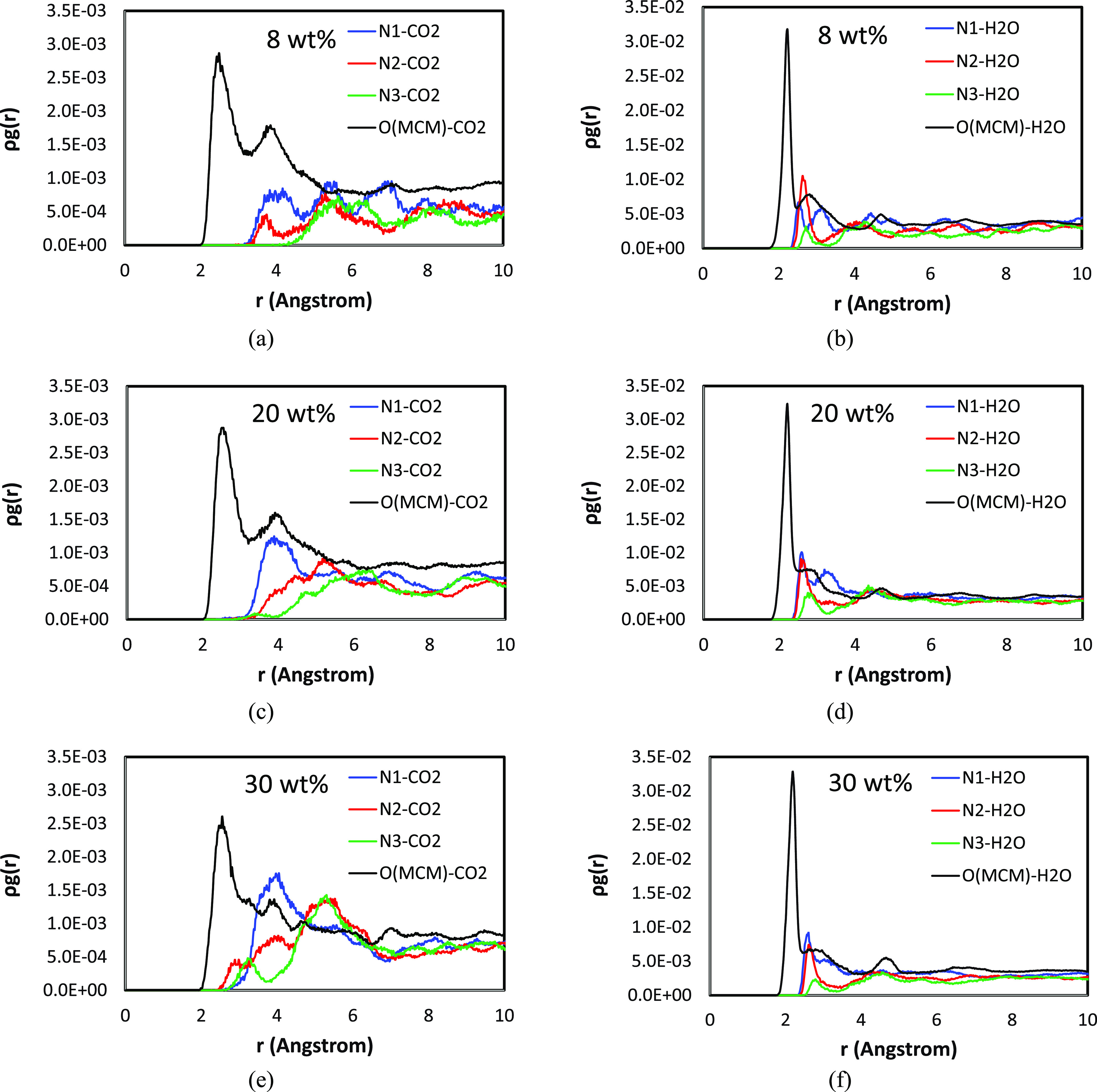
Pair correlation analysis of amine-CO_2_, MCM-41-CO_2_, amine-H_2_O, and MCM-41-H_2_O pairs in PEI/MCM systems (PEI/MCM-*L*-288-72, *L* = 8, 20, and 30% HB-PEI content): (a, b) 8% HB-PEI contents; (c, d) 20% HB-PEI contents; and (e, f) 30% HB-PEI contents. N1, N2, and N3 denote the primary, secondary, and tertiary amines. The numbers of CO_2_ molecules and H_2_O molecules are 72 and 288, respectively.

**Figure 9:**
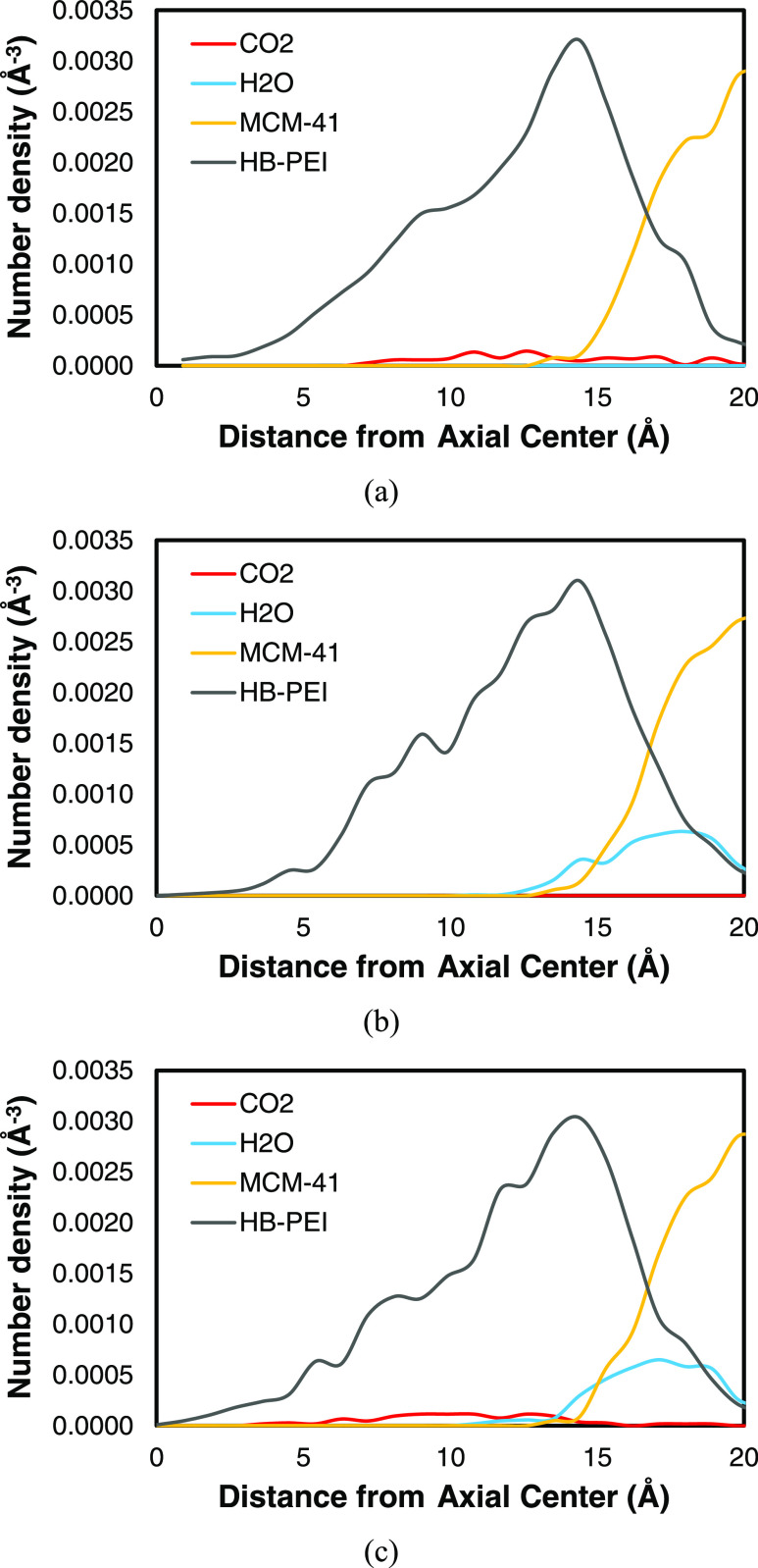
Cylindrical density distribution of CO_2_, H_2_O, HB-PEI, and MCM-41 in PEI/MCM systems with 34 wt % HB-PEI content: (a) PEI/MCM-34-0-72, (b) PEI/MCM-34-288-0; and (c) PEI/MCM-34-288-72.

We first analyzed these pair correlations among the pure CO_2_ cases, pure H_2_O cases, and the mixed CO_2_/H_2_O cases (Figure 7) and the change of these pair correlations as a function of the HB-PEI content for the mixed species case (Figure 9). We also characterized the cylindrical density distribution of the four molecular species as a function of the distance from the central axial line of the pore (Figure 8).

#### 3.3.1 Amine-CO_2_ and MCM-CO_2_ Pair Correlations

Since the reactions of CO_2_ with amine groups require proximity between the two species, we first analyzed the amine-CO_2_ pair correlation, *ρg*(*r*)(N–CO_2_), in the PEI/MCM systems.

Figure 7a shows the correlation of CO_2_ molecules with the MCM-41 pore surface and amine groups in the absence of H_2_O molecules in PEI/MCM-34-0-72. We observed that *ρg*(*r*)(O(MCM)–CO_2_) had a peak at *r* = ∼2.5 Å, indicating a direct correlation between CO_2_ molecules and the MCM-41 pore surface. For amine-CO_2_ pairs, the N1-CO_2_ and N2-CO_2_ pairs had direct correlations starting from *r* = ∼3.0 Å, while the N2-CO_2_ and N3-CO_2_ pairs had strong indirect correlations at *r* = ∼5.2 Å. The indirect correlation is induced by other direct correlations, such as N1-CO_2_ and N2-CO_2_ pair correlations at ∼2.5 Å < *r* < ∼4.5 Å. We previously reported such indirect correlations of CO_2_ with N2 and N3 in our study on CO_2_ in a HB-PEI membrane,^1^ which is similar to the indirect correlations observed in the PEI/MCM system of this study. Comparing the details in the amine-CO_2_ pair correlations, it appears that the amine-CO_2_ pair correlations in the PEI/MCM system are weakened due to the strong interaction between amines and MCM-41.

We then analyzed the amine-CO_2_ pair correlations in the PEI/MCM systems in the presence of H_2_O molecules in PEI/MCM-34-288-72, and it appears that the presence of H_2_O affects the amine-CO_2_ pair correlations due to the strong molecular interactions of H_2_O with amines and MCM-41, as displayed in Figure 2b,d. Because the silanol groups of MCM-41 are associated with H_2_O molecules, a portion of the amine groups, specifically the primary amine (N1) groups, are dissociated from the MCM-41 pore surface. These N1 groups can then associate with more CO_2_ molecules, resulting in a significant increase in the N1-CO_2_ pair correlation, as shown in Figure 7c. In contrast, the N2-CO_2_ and N3-CO_2_ correlations decrease correspondingly, as the number of CO_2_ molecules in the simulation remains constant.

We also investigated the effect of HB-PEI content on the distribution of CO_2_ molecules in the presence of H_2_O in PEI/MCM-*L*-288-72 (*L* = 8, 20, and 30), as shown in Figure 8a,8c,8e. As discussed in Figure 7, all amines have a correlation with CO_2_ at 5 Å < *r* < 6 Å, which is likely an indirect correlation induced by the direct correlation of CO_2_ with primary and secondary amines. In other words, the CO_2_ molecules in the vicinity of primary and secondary amine groups at 3.0 Å < *r* < 4.5 Å are also located near other amine groups at 5 Å < *r* < 6 Å, as confirmed in Figure 1a.

It is worth noting that for the 8 and 20 wt % HB-PEI contents (as shown in Figures 8a and 8c, respectively), the MCM-41-CO_2_ pair correlation is overwhelmingly strong compared to the amine-CO_2_ pair correlations. Considering that the number of amine groups is not statistically sufficient with CO_2_ molecules, the CO_2_ molecules need to gather at the HB-PEI/MCM-41 interface to gain more stabilizing molecular interaction. Therefore, the low levels of HB-PEI content allow more correlation of the CO_2_ molecules with the MCM-41 surface. In contrast, for the 30 wt % HB-PEI content (Figure 8e), the MCM-41 pore surface is occupied by the amine groups of HB-PEI with strong binding energies (−31.49, −31.26, and −31.42 kcal/mol for SiO_2_-N1, SiO_2_-N2, and SiO_2_-N3 pairs, respectively, as shown in Figure 2a). As a result, the MCM-41 pore surface has a weaker correlation with CO_2_ adsorption.

#### 3.3.2 Amine-H_2_O and MCM-H_2_O Pair Correlations

The correlations of H_2_O molecules with amine groups and MCM-41 are other interesting features in the PEI/MCM systems because the strong molecular interactions of H_2_O with other components play a role in the structure-determining factor.

Figure 7b shows the correlation of H_2_O molecules with the MCM-41 pore surface and amine groups in the absence of the CO_2_ molecules in PEI/MCM-34-288-0. We observed that *ρg*(*r*)(O(MCM)–H_2_O) had a peak at *r* = ∼2.2 Å, indicating a direct correlation between H_2_O molecules and the MCM-41 pore surface. Unlike the amine-CO_2_ pairs, the amine-H_2_O pair correlations showed the first peak at *r* = ∼2.7 Å, indicating that they are all direct correlations and are relatively stronger than the amine-CO_2_ correlations. Another point to note in Figure 7b is that the N1-H_2_O and N2-H_2_O pairs have stronger correlations compared to the N3-H_2_O pair due to the number of attached hydrogens.

Next, by analyzing the amine-H_2_O pair correlations in the PEI/MCM system in the presence of CO_2_ molecules in PEI/MCM-34-288-72, we found that the amine-H_2_O pair correlations are reduced, and the MCM-H_2_O correlation is nearly the same in the presence of CO_2_, which is attributed to the competition with CO_2_ for the amine groups. However, it is noted from the comparison between Figure 7b,7d that the overall feature of the correlations of H_2_O with other components in the system is nearly the same because the structure of the system is driven by the strong molecular interaction of H_2_O with other components.

The features in Figure 7b,7d are also revealed from the simulations of PEI/MCM-*L*-288-72 (*L* = 8, 20, and 30 wt % HB-PEI content) with 72 CO_2_ and 288 H_2_O in Figure 8b,8d,8f, showing that the hydrophilic amine groups and silanol groups are associated with H_2_O molecules at 2.0 Å < *r* < 5.5 Å. It is confirmed that all of the correlations of H_2_O are almost insensitive to the HB-PEI content. First, the MCM-H_2_O correlation at *r* = ∼2.2 Å is overwhelming compared to any other correlations in the system, regardless of the HB-PEI content variation due to the strong MCM-H_2_O molecular interaction. On the other hand, the amine-H_2_O correlations at *r* = ∼2.6 Å are also insensitive to the HB-PEI content, in which the N1-H_2_O and N2-H_2_O pair correlations have similar intensity, while the N3-H_2_O pair correlation has the weakest intensity. Thus, it is concluded that the amine-H_2_O interactions are affected by hydrogen-bonding interaction and steric hindrance.

#### 3.3.3 Cylindrical Density Distribution

Figure 9 presents the cylindrical density distributions of four molecular species as a function of the distance from the axial center of the pore for three systems, such as PEI/MCM-34-0-72, PEI/MCM-34-288-0, and PEI/MCM-34-288-72.

First, it is commonly observed that the HB-PEI phase fills the pore of MCM-41, with its maximum number density found at 13 Å < *r* < 15 Å. By considering the surface of MCM-41 at 13 Å < *r*, the density profiles of HB-PEI indicate that the HB-PEI molecules are adsorbed on the pore surface, which aligns well with the newly optimized force field for the strong interaction between amine and the pore surface (Figure 2a). Second, in the absence of H_2_O, CO_2_ molecules are distributed throughout the HB-PEI phase and at the surface of the MCM-41 pores (Figure 9a). Conversely, in the absence of CO_2_, H_2_O molecules are concentrated near the surface of MCM-41 (Figure 9b). These observations are consistent with the pair correlation analysis (Figures 7a,7b) and the newly introduced force field parameters. Lastly, Figure 9c illustrates the density profile for the case of the CO_2_/H_2_O mixed gas in the PEI/MCM system, where the two characteristics individually reported in Figure 9a,9b are observed together: CO_2_ molecules are evenly distributed, and H_2_O molecules gather at the surface of MCM-41. Here, it is important to note that H_2_O molecules drive CO_2_ molecules away from the surface of MCM-41 due to the differences in their molecular interactions with MCM-41, as discussed in Figures 2b and 7c,7d. Consequently, it can be concluded that the presence of moisture leads to greater availability of CO_2_ molecules in the HB-PEI phase, which is beneficial for CO_2_ capture.

### 3.4 Diffusion of CO_2_ and H_2_O

**Figure 10:**
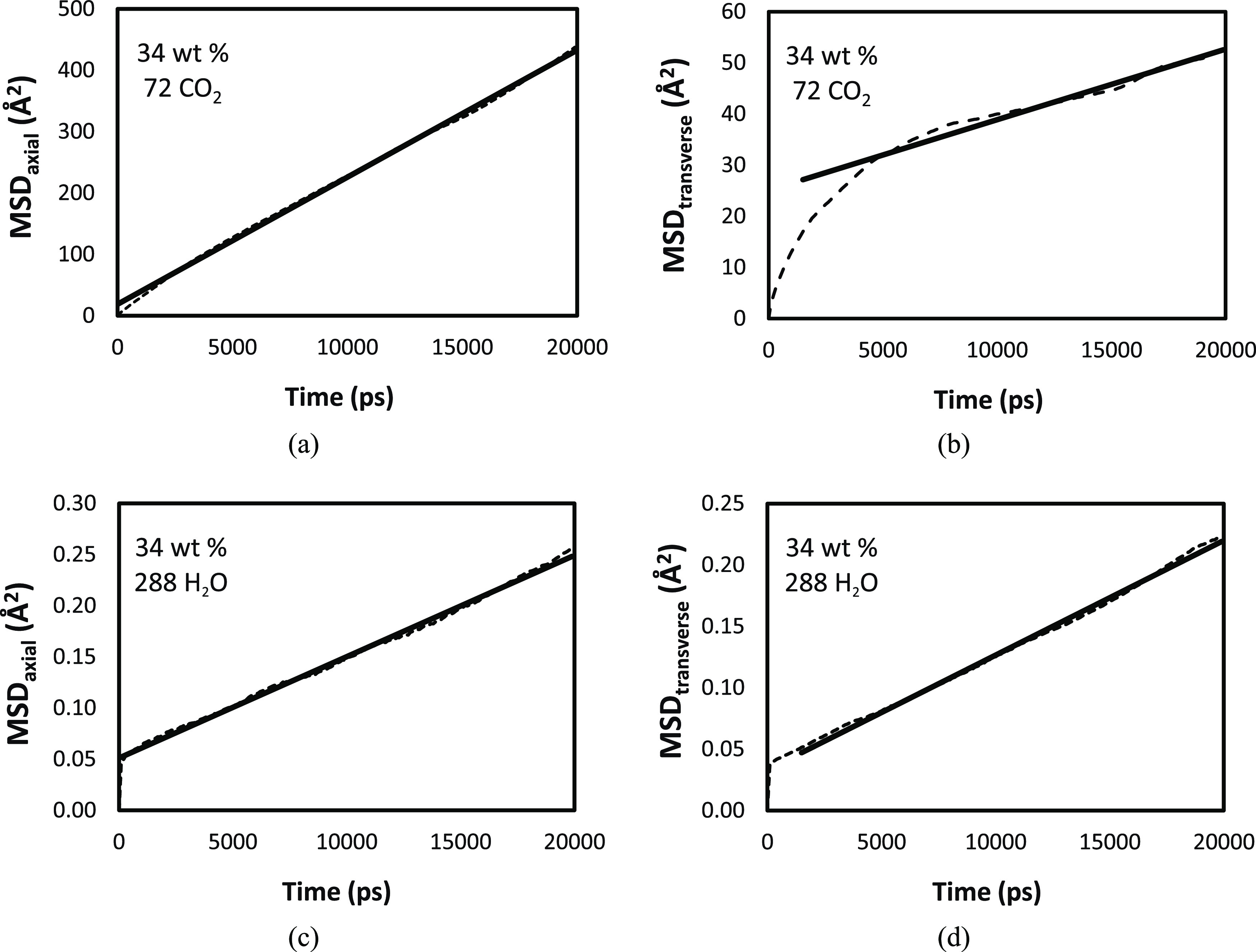
MSD of gas molecules in PEI/MCM with 34 wt % HB-PEI content (PEI/MCM-34-288-72): for CO_2_ (a) in the axial direction; (b) in the transverse direction, and for H_2_O (c) in the axial direction; (d) in the transverse direction. The diffusion coefficients in Tables 2 and 3 are calculated from the linear regimes of the variation of MSD as a function of time.

Efficient CO_2_ capture within the PEI/MCM system relies heavily on the transport of CO_2_ and H_2_O molecules, as poor transport can impede the adsorption–desorption process and negatively impact the overall performance. To quantify the transport behavior, we analyzed the mean square displacement (MSD) of CO_2_ and H_2_O molecules over time, which is shown in Figure 10 as an example since all PEI/MCM systems have similar behavior of MSD. Given that the molecules are confined within a pore of MCM-41, it is desirable to examine the diffusion of CO_2_ and H_2_O with respect to two distinct directions such as axial and transverse directions. Starting from the overall MSD
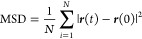
6where ***r***(*t*) and ***r***(0) denote the positions of particle *i* at time *t* and the beginning, *N* denotes the number of particles the overall MSD can break down to three components since ***r***(*t*) **–*****r***(0) = (*r*_*x*_(*t*) – *r*_*x*_(0), *r*_*y*_(*t*) – *r*_*y*_(0), *r*_*z*_(*t*) – *r*_*z*_(0))
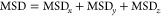
7

Accordingly, from the overall diffusion coefficient (*D*)
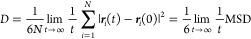
8we can write the axial diffusion coefficient (*D*_axial_) along the pore direction (*z*-axis direction) and transverse diffusion coefficient (*D*_transverse_) perpendicular to the pore direction (in *x*-*y* plane)
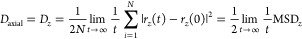
9
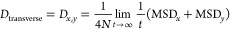
10Using this approach, we calculated diffusion coefficients as summarized in Table 2 for the cases of pure CO_2_ and H_2_O, and in Table 3 for the cases of CO_2_/H_2_O mixed gas, through PEI/MCM systems with 34 wt % HB-PEI content.

**Table 2:** Self-Diffusion Coefficients of Pure CO_2_ and Pure H_2_O in PEI/MCM with 34 wt % HB-PEI Content

	CO_2_			H_2_O	
number of CO_2_	*D*_axial_ (×10^–5^ cm^2^/s)	*D*_transverse_ (×10^–5^ cm^2^/s)	number of H_2_O	*D*_axial_ (×10^–8^ cm^2^/s)	*D*_transverse_ (×10^–8^ cm^2^/s)
12	0.00009	0.00006	72	0.01971	0.01369
24	0.00055	0.00031	144	0.02238	0.01765
36	0.00529	0.00471	288	0.05275	0.02457
48	0.00985	0.00687			
72	0.06256	0.00703			

**Table 3:** Self-Diffusion Coefficients of CO_2_/H_2_O Mixed Gas in PEI/MCM with 34 wt % HB-PEI Content

	CO_2_		H_2_O	
number of H_2_O–number of CO_2_	*D*_axial_ (×10^–5^ cm^2^/s)	*D*_transverse_ (×10^–5^ cm^2^/s)	*D*_axial_ (×10^–8^ cm^2^/s)	*D*_transverse_ (×10^–8^ cm^2^/s)
72–72	0.1351	0.00324	0.0491	0.0223
288–72	0.1482	0.00625	0.0505	0.0392

First, it is noted that the diffusion coefficients in Tables 2 and 3 are significantly smaller than those of CO_2_ (*D*(CO_2_) = 1.093 × 10^–5^ cm^2^/s) and H_2_O (*D*(H_2_O) = 0.687 × 10^–5^ cm^2^/s) in the HB-PEI bulk phase. This significant reduction of CO_2_ diffusion in the HB-PEI-loaded MCM-41 can be ascribed to the molecular mobility suppression of HB-PEI due to the strong interaction of amine groups with MCM-41, while that of H_2_O diffusion is due to the strong interaction of H_2_O with MCM-41. The second point revealed in Tables 2 and 3 is that *D*_axial_ is greater than *D*_transverse_. We think this is primarily due to the nanoconfinement effect of the pore with a diameter of ∼3 nm in transverse direction, compared to the semi-infinite dimension of the pore in the axial direction, meaning that the difference between *D*_axial_ and *D*_transverse_ would be decreased as a function of the pore diameter.

Another noteworthy observation from Table 2 is the increase in the diffusion coefficient of pure gas in the PEI/MCM system with respect to the CO_2_ and H_2_O concentrations. This finding suggests that a higher concentration of gas molecules enables a greater number of molecules to exist in the nonadsorbed state within the systems.

Lastly, we analyzed axial and transverse molecular diffusion of the CO_2_/H_2_O mixture in PEI/MCM-34-72-72 and PEI/MCM-34-288-72, as summarized in Table 3. In the mixed gas simulations, we observed a significant increase in *D*(CO_2_) in the presence of H_2_O.^48−51^ As discussed in the pair correlation section, this increase in *D*(CO_2_) arises from the interaction between H_2_O and silanol groups, which effectively screen the interaction of CO_2_ molecules with the pore surface of MCM-41. Therefore, in the presence of H_2_O, more CO_2_ molecules are dissociated from the pore surface and available in the nonadsorbed state, leading to an increase in *D*(*CO*_2_).

In contrast, we observed a relatively small change in *D*(H_2_O) in the presence of CO_2_. We believe this is because *D*(H_2_O) is governed by the strong molecular interactions between H_2_O and silanol groups such that the presence of CO_2_ molecules does not affect the status of H_2_O molecules. Consequently, the effect of the H_2_O concentration on *D*(CO_2_) was the only discernible trend observed in Table 3. Overall, our diffusion analysis suggests that increasing the humidity of the CO_2_ gas can enhance the transport of CO_2_ through PEI/MCM systems by pushing the CO_2_ molecules away from the silanol groups using H_2_O molecules.

## 4 Conclusions

In this study, we investigated the distribution and transport of CO_2_ in the HB-PEI-loaded MCM-41 system by using molecular dynamics simulation methods. The force field parameters were newly developed using DFT modeling to describe the interactions among the components in the system such as CO_2_, H_2_O, HB-PEI, and MCM-41.

The study also revealed that the most thermodynamically favorable HB-PEI loading was around 34 wt %, where HB-PEI fully covers the MCM-41 pore surface. Using pair correlation analysis, the study found that H_2_O molecules dominate the molecular adsorption in HB-PEI-loaded MCM-41 due to their strong affinity with silanol groups on MCM-41 and amine groups in HB-PEI, resulting in competition with CO_2_. Therefore, the HB-PEI content significantly affects the molecular distribution of CO_2_ in the PEI/MCM system, while the distribution of H_2_O is nearly insensitive to the variation of the HB-PEI content.

The study found that the self-diffusion coefficient of CO_2_, *D*(CO_2_) in the PEI/MCM system, was significantly lower than that in the pure HB-PEI membrane due to the attractive interaction of CO_2_ molecules with silanol groups on the MCM-41 pore surface. However, *D*(CO_2_) increased in the PEI/MCM system with 34 wt % HB-PEI content as a function of the CO_2_ concentration because more CO_2_ molecules can be free from the silanol groups and become associated with the HB-PEI phase of the PEI/MCM system.

The study also found that the self-diffusion coefficient of H_2_O, *D*(*H*_2_*O*) in the PEI/MCM system, was considerably lower than that in the pure HB-PEI membrane due to the strong interaction of H_2_O with silanol groups of MCM-41. However, *D*(H_2_O) increased as a function of H_2_O concentration because more H_2_O molecules could be distributed without interacting with the silanol groups.

When CO_2_ and H_2_O were present together in the PEI/MCM system, we observed a drastic increase in the self-diffusion coefficient of CO_2_ compared to that in the dry CO_2_ cases because the silanol groups were not available for CO_2_, as they were primarily associated with H_2_O molecules. In contrast, the diffusion coefficient of H_2_O remained relatively unchanged compared to the pure H_2_O cases due to the overwhelmingly strong interaction of H_2_O with silanol groups.

Overall, the study concluded that the presence of H_2_O molecules increases the CO_2_ correlation with the amine groups and CO_2_ transport and further makes the bicarbonate formation mechanism available, which means that the presence of H_2_O enhances the CO_2_ capture in the HB-PEI-loaded MCM-41.

## Supporting Information Available

Table for newly optimized force field parameters and a figure for adsorptive binding energy curves Lorentz–Berthelot standard mixing rules (PDF)
