# Supplementary material for: Distribution and Transport of CO2 in Hyperbranched Poly(ethylenimine)-Loaded MCM-41: A Molecular Dynamics Simulation Approach
Source: ACS Appl Mater Interfaces. 2023 Sep 8;15(37):43678–90. doi: 10.1021/acsami.3c07040 (PMC10520917; doi:10.1021/acsami.3c07040)
Supplement: Supplementary file 1 — am3c07040_si_001.pdf [file am3c07040_si_001.pdf]

Supporting Information

Distribution and Transport of CO_2 in Hyperbranched Poly(ethyleneimine)-loaded MCM-41: Molecular Dynamics Simulation Approach

Junhe Chen,[1] Hyun June Moon,[2] Kyung Il Kim,[1,2] Ji Il Choi,[1] Pavithra Narayanan,[2] Miles A. Sakwa-Novak,[3] Christopher W. Jones,[2,*] and Seung Soon Jang[1,4,*]

[1] Computational NanoBio Technology Laboratory, School of Materials Science and Engineering, Georgia Institute of Technology, 771 Ferst Drive NW, Atlanta, GA 30332-0245, USA

[2] School of Chemical & Biomolecular Engineering, Georgia Institute of Technology, 311 Ferst Drive NW, Atlanta, GA 30332-0100, USA

[3] Global Thermostat LLC, 10275 E106th Ave, Brighton, CO 80601

[4] Strategic Energy Institute, Georgia Institute of Technology, Atlanta, GA, 30332, USA

[*] Corresponding author:

Christopher Jones cjones@chbe.gatech.edu orcid.org/0000-0003-3255-5791

Seung Soon Jang seungsoon.jang@mse.gatech.edu orcid.org/0000-0002-1920-421X

Keywords: CO_2 Capture, Hyperbranched Poly(ethylenimine), MCM-41, Force Field, Molecular Dynamics Simulation

Table S1. Lennard-Jones parameters (r_0 and D) for *o*ff-diagonal van der Waals Interaction

Atom A	Atom B	r_0 (Å)	D (kcal/mol)
C_1	C_3	3.4098	0.09734
C_1	N_3	3.0474	0.13645
C_1	H_	3.8332	0.04576
C_1	H___A	4.0868	0.00319
C_1	H__OH	3.4794	0.45043
C_1	O_3	2.0481	0.22399
C_1	Si_3	2.4770	1.56320
O_2	C_3	3.3252	0.11176
O_2	N_3	3.5118	0.11451
O_2	H_	2.4541	0.52264
O_2	H___A	2.7926	0.82499
O_2	H__OH	3.1332	0.00069
O_2	O_3	2.0622	0.68438
O_2	Si_3	3.8709	1.24816
O_F3C	C_3	3.8428	0.20793
O_F3C	N_3	3.3747	0.18185
O_F3C	H_	2.4452	0.88539
O_F3C	H___A	3.3430	1.69864
O_F3C	H__OH	2.0716	0.00141
O_F3C	O_3	2.4572	0.15767
O_F3C	Si_3	3.1936	0.11923
H_F3C	C_3	1.9368	0.03100
H_F3C	N_3	1.4714	0.02689
H_F3C	H_	1.8156	0.01248
H_F3C	H___A	1.6959	0.00100
H_F3C	H__OH	1.5035	0.76496
H_F3C	O_3	2.4315	0.00119
H_F3C	Si_3	3.3844	5.79225
Si_3	N_3	3.8434	0.70425
Si_3	C_3	3.4847	0.60690
Si_3	H_	3.5426	0.55796
Si_3	H___A	3.1733	1.33443
O_3	N_3	1.9351	0.43429
O_3	C_3	3.2034	0.07385
O_3	H_	2.8516	0.45291
O_3	H___A	2.5809	1.26660
H__OH	N_3	1.1310	0.59380
H__OH	C_3	3.3812	0.02434
H__OH	H_	1.5796	0.09989
H__OH	H___A	1.1491	0.14968

Table S2. Lennard-Jones parameters (r_0 and D) for diagonal van der Waals Interaction

Atom	r_0 (Å)	D (kcal/mol)
O_F3C	3.5532	0.18480
H_F3C	0.9000	0.01000
C_3	3.8983	0.09510
N_3	3.6621	0.07740
H_	3.1950	0.01520
H___A	3.1950	0.00010

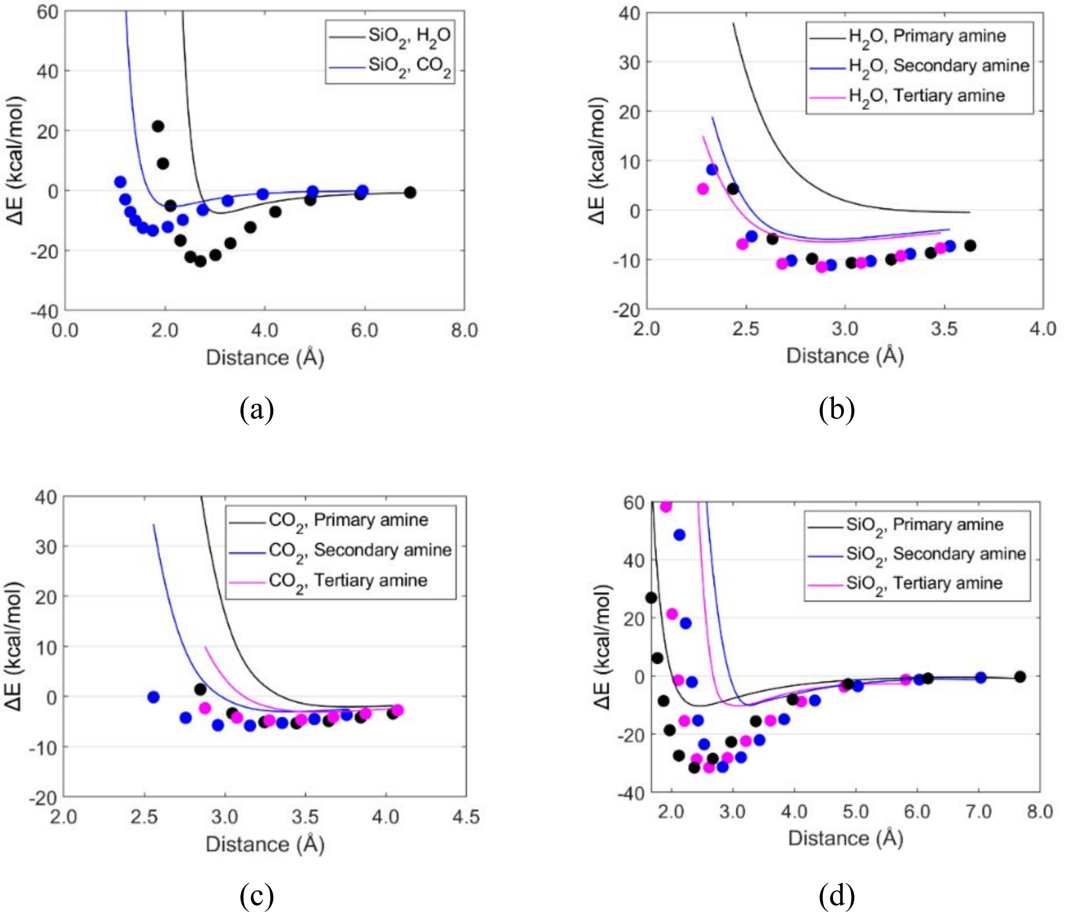

Figure S1. Adsorption binding energies calculated using Lorentz-Berthelot standard mixing rules (solid curves) in comparison to those from DFT calculations (colored circles): (a) SiO_2–amines; (b) SiO_2–water and CO_2; (c) CO_2–amines; (d) H_2O–amines.
